# Artificial Intelligence in Higher Education: The Impact of Need Satisfaction on Artificial Intelligence Literacy Mediated by Self-Regulated Learning Strategies

**DOI:** 10.3390/bs15020165

**Published:** 2025-02-02

**Authors:** Kai Wang, Wencheng Cui, Xue Yuan

**Affiliations:** 1 Center for Teacher Education Research, Beijing Normal University, Beijing 100091, China; wangkai1991@bnu.edu.cn; 2 School of Education, Minzu University of China, Beijing 100081, China; 3 Faculty of Law and Criminology, Vrije Universiteit Brussel (VUB), Pleinlaan 2, 1050 Elsene, Belgium; xue.yuan@vub.be

**Keywords:** artificial intelligence literacy, artificial intelligence in higher education, self-regulated learning strategies, self-determination theory, need satisfaction

## Abstract

Artificial intelligence (AI) technologies have profoundly influenced both professional environments and personal lives. In the rapidly developing sector of AI education, fostering essential AI literacy among university students has become vital. Nevertheless, the factors that determine AI literacy remain insufficiently defined. This research, grounded in self-determination theory (SDT), seeks to investigate the relationships among three components: the fulfillment of university students’ three psychological needs, self-regulated learning strategies (SRLSs), and AI literacy. The aim is to enhance human capital efficiency and prepare students to tackle future workplace challenges effectively. To examine these connections, a cross-sectional survey was administered to 1056 university students. The findings reveal that satisfying the three psychological needs—perceived autonomy, competence, and relatedness—plays a pivotal role in advancing AI literacy among university students. Additionally, four SRLSs—cognitive engagement, metacognitive knowledge, resource management, and motivational beliefs—acted as mediators between these psychological needs and AI literacy. Consequently, this study not only enhances our understanding of the psychological and behavioral development of university students during their engagement with AI education but also provides theoretical support and practical guidance for fostering their AI literacy.

## 1. Introduction

Artificial intelligence (AI) describes the capability of computer systems to carry out activities that traditionally required human cognitive abilities, including learning, reasoning, problem-solving, sensory perception, and understanding language (Stuart & Peter, 2016). Compared to previous generations of information technology, artificial intelligence has achieved significant technical advances over the past decade, exerting a profound impact on various areas of society, including education (Su et al., 2023). AI in education (AIED) has become an evolving multidisciplinary field that utilizes artificial intelligence to enhance and streamline teaching, learning processes, design, and evaluations (Ouyang & Jiao, 2021). The interconnection between higher education and AI technology remains significant, requiring students to adapt swiftly to the evolving needs of contemporary society (Crompton & Burke, 2023). Universities encourage all students, beyond those specializing in computer science or actively involved in AI technology development, to develop the skills necessary for managing AI in their future careers, regardless of their professional area (Ng et al., 2021; Southworth et al., 2023). However, the widespread deployment of AI also presents several significant challenges. A primary concern involves the potential misuse of AI technologies (Ivanov, 2023). Several studies indicate that the compulsive use of online platforms may lead to reduced face-to-face social interactions, disrupted sleep patterns, and a general decline in mental health, often exacerbating symptoms of anxiety and depression (Alonzo et al., 2021; Hussain & Griffiths, 2021). The improper use of AI technologies could similarly exacerbate these risks. This emphasizes the requirement for a new form of literacy—AI literacy.

AI literacy constitutes a comprehensive concept that not only involves the comprehension of AI systems but also the responsible and proficient utilization of these technologies. Additionally, it includes the application of critical thinking in their design and execution. According to Ng et al. (2021), this type of literacy is defined as the capability to critically understand, assess, and implement AI technologies without the requirement of independently developing AI models. AI literacy has become essential globally as AI applications have proliferated (Luckin & Holmes, 2016; Ng et al., 2021). However, despite the increasing global presence of AI, its integration into non-STEM undergraduate and graduate education has remained limited and inconsistent (Cantú-Ortiz et al., 2020; Dai et al., 2020), leaving numerous students inadequately prepared in AI literacy.

Research in AI-driven educational technology has primarily focused on the development of systems or applications (Song & Wang, 2020; Zawacki-Richter et al., 2019). AI algorithms (Chame et al., 2019), conversational agents (X. Yang & Aurisicchio, 2021), and AI-assisted decision-making processes (De Vreede et al., 2021) have all drawn upon self-determination theory (SDT). Recently, Ryan and Deci (2020), the originators of SDT, recommended that SDT research concentrate on the development of educational technologies. AI technologies possessed significant anthropomorphic characteristics, allowing them to interact with students. University students were expected to demonstrate initiative, formulate strategies, interact with these technologies, and actively participate in their educational activities (Xia et al., 2023b). Additionally, compared to K-12 schooling, learners in higher education enjoyed enhanced autonomy due to the reduced external oversight of their learning processes (Jansen et al., 2019). This heightened independence required students to exert greater control over their own learning, thereby making self-regulated learning (SRL) increasingly essential (Luo & Zhou, 2024). SRL extends beyond being solely a psychological trait or an academic competency; it embodies a self-initiated approach through which students harness their cognitive abilities to develop and implement effective learning strategies (Zimmerman, 2002). According to Pintrich (1999), SRL entails students actively directing their own educational activities by implementing various strategies, including cognitive, metacognitive, motivational, and resource management techniques. This proactive approach allows learners to effectively control and optimize their learning experiences, fostering greater academic achievement and personal growth. In recent years, research in learning sciences has increasingly acknowledged the potential of leveraging intelligent learning technologies to analyze and support SRL. Learners’ SRL was influenced by motivation (B. Bai et al., 2024). According to SDT, fulfilling fundamental psychological needs—namely, autonomy, competence, and relatedness—enhances intrinsic motivation and promotes ongoing learning behaviors (Ryan & Deci, 2000). When individuals’ fundamental psychological requirements are fulfilled, they tend to develop greater autonomy and exhibit increased participation in SRL activities (Liu et al., 2014; Sierens et al., 2009).

To the best of our knowledge, while SDT and SRL have been extensively studied within the context of AI education, few studies have investigated their intersection with AI literacy. Addressing this gap in the literature, the present study aims to provide a comprehensive explanation of how the psychological needs of autonomy, competence, and relatedness in SDT influence the four dimensions of students’ AI literacy—awareness, usage, assessment, and ethics—and examines the mediating role of SRLSs in this relationship.

## 2. Literature Review

### 2.1. Understanding AI Literacy from a Self-Determination Theory Perspective

AI literacy is similar to computer and digital literacy, involving the ethical identification, use, and evaluation of AI products without requiring deep technical knowledge (Ng et al., 2021). That is, being able to use AI products effectively and with ethically defined AI literacy (B. Wang et al., 2023). Currently, the realm of AI is continuously expanding and being integrated into diverse sectors. Consequently, pinpointing the exact competencies that characterize an individual with AI literacy has become increasingly complex. In fact, Moore (2011) argued that AI literacy ‘should not be thought of as an isolated set of skills but as an essential strategy for most learning objectives’. Building upon this foundation, B. Wang et al. (2023) examined the intricate interplay between digital and AI technologies by leveraging Wilson et al.’s (2015) technological-cognitive-ethical model model alongside the KSAVE framework within the Information and Communication Technologies (ICT) domain. They introduced a comprehensive theoretical model for AI literacy that comprises four key components: awareness (the capability to identify and comprehend AI technologies while utilizing AI-driven applications), use (the proficiency to effectively implement and employ AI technologies to accomplish tasks efficiently), evaluation (the skill to critically analyze, choose, and assess AI applications and their resulting outcomes), and ethics (the understanding of the responsibilities and potential risks linked to the deployment and use of AI technologies). In the context of human–AI interactions (HAIIs), the AI Literacy Scale (AILS) is designed for general populations. The four dimensions—awareness, usage, ethics, and evaluation—provide a comprehensive framework that aligns with the established constructs of AI literacy found in multiple other scales. These dimensions address key aspects of AI literacy that are essential for students to navigate an increasingly AI-driven world. Other AI literacy studies, such as those by H. Zhang et al. (2024) and Ng et al. (2024), focus primarily on middle school or secondary education students. In contrast, the SNAIL (Self-Reported Non-Expert AI Literacy) scale, which targets university students, examines dimensions such as technical understanding, critical appraisal, and practical application, but notably omits a focus on ethics. Given these considerations, selecting B. Wang et al. (2023)’s AILS for this study is highly appropriate.

SDT emphasizes the critical role of intrinsic motivation, which is driven by the inherent desire to engage in activities for personal fulfillment rather than external rewards (Ryan & Deci, 2000). This theory posits that satisfying basic psychological needs fosters intrinsic motivation, thereby supporting sustained and meaningful learning behaviors (Ryan & Deci, 2000). Specifically, autonomy, competence, and relatedness are essential for creating an environment that fosters motivation and engagement in learning. Existing research has demonstrated SDT’s effectiveness in various educational contexts, including AI-enhanced learning environments. For example, Chiu et al. (2023) found that SDT-based instructor support significantly enhanced student motivation and learning outcomes when using AI chatbots in teaching. Similarly, Xia et al. (2023b) explored the role of SDT in self-directed learning in AI-enhanced environments, highlighting the importance of proficiency in both English and AI for successful learning. In contrast to SDT, other motivational or learning theories, such as Bandura’s Social Learning Theory, place greater emphasis on external social factors and behavioral modeling, rather than directly addressing the internal drivers of motivation (Bandura, 1977). Furthermore, while Sweller’s Cognitive Load Theory highlights the cognitive processes involved in learning new technologies, it overlooks the motivational dynamics that affect students’ persistence and engagement during the learning process (Sweller, 1988). These findings suggest that SDT offers a robust framework for fostering intrinsic motivation and active engagement in AI learning (Taranto & Buchanan, 2020; Xia et al., 2022), particularly in dynamic and complex domains like AI (Long & Magerko, 2020; Ryan & Deci, 2017; Warschauer & Matuchniak, 2010).

Despite the promise of SDT in educational settings, there is a notable gap in the literature regarding its application to AI literacy. Currently, there is a lack of empirical evidence directly linking the fulfillment of psychological needs, as outlined in SDT, to specific elements of AI literacy. While SDT has been widely applied to understand various dimensions of digital literacy, the relationship between the satisfaction of learners’ psychological needs and AI literacy remains underexplored. Studies grounded in SDT suggest that fulfilling learners’ three primary psychological needs—autonomy, competence, and relatedness—can significantly enhance multiple facets of their digital literacy (Chiu et al., 2022). Recent studies have begun to explore the relationship between psychological needs and AI literacy. For instance, Shen and Cui (2024) found that autonomy and competence positively influenced college students’ AI literacy, indicating that meeting these psychological needs may enhance students’ AI competencies. Additionally, Q. Yang et al. (2024) confirmed that emotional engagement fully mediates the relationship between autonomy, competence, and AI literacy. However, these studies have yet to examine how autonomy, competence, and relatedness influence the different aspects of AI literacy, such as awareness, usage, evaluation, and ethics. This gap in the literature underscores the need for a more nuanced exploration of the ways in which SDT’s core psychological needs contribute to AI literacy development.

### 2.2. Self-Regulated Learning Strategies

Pintrich (1999) characterizes SRL as the proactive management of one’s own educational activities through various strategies. These include cognitive strategies such as retelling, exposition, organizing information, and engaging in critical thinking; metacognitive strategies like planning, monitoring progress, and adjusting approaches; motivational strategies including self-motivation, building connections, and positive self-talk; and resource management strategies such as time management, optimizing learning environments, collaborative learning, and seeking assistance.

Research exploring the connections between the three fundamental psychological needs and SRL indicates that these needs significantly influence SRL processes. For example, J. Zheng et al. (2020) discovered that providing autonomy support directly enhances students’ SRL abilities and their orientation towards academic goals, which subsequently reduces levels of academic stress. The perception of competence is equally crucial; students who believe in their own capabilities are more likely to consistently employ SRLSs and exhibit higher intrinsic motivation (Pelikan et al., 2021). Additionally, when learners perceive the relevance of the knowledge they are acquiring, they tend to show increased interest, heightened motivation, and deeper engagement in their educational activities (Grolnick & Raftery-Helmer, 2015; Zhou et al., 2021).

Studies have shown that SRLSs exert both direct and indirect influences on students’ literacy competencies (Anthonysamy et al., 2020; Chen et al., 2021; Z. Zhang et al., 2023). For example, Anthonysamy et al. (2020) confirmed three hypotheses, suggesting that metacognitive knowledge, resource management, and motivational beliefs significantly and positively influence digital literacy. These findings imply that SRLSs play a critical role in fostering key competencies necessary for navigating the rapidly evolving digital landscape. In another study, Z. Zhang et al. (2023) found that teachers’ self-efficacy significantly enhances their ICT literacy, with online SRLSs serving as an intermediary. Additionally, Chen et al. (2021) investigated the impact of social media on adolescents’ digital reading literacy within a cross-cultural framework, discovering that SRL components, such as metacognitive strategy knowledge, reading enjoyment, and self-concept, mediated this relationship.

Recent studies have also examined the potential synergy between SRL theory and AI (Jin et al., 2023; Molenaar, 2022; Molenaar et al., 2023). However, the current body of research on the relationship between AI and SRL has primarily focused on how AI supports and enhances SRL. For example, Lim et al. (2023) explored the application of rule-based AI systems for real-time measurement and support of students’ SRL. Their findings revealed that personalized scaffolding, facilitated by real-time analysis, significantly enhances students’ SRL. Similarly, several studies have investigated the role of SRLSs in AIED. For instance, Su (2020) emphasized that SRL-based adaptive scaffolding strategies are crucial for the effective implementation and success of computer software learning. Despite these contributions, a significant gap remains in the literature regarding how SRLSs specifically influence AI literacy. Furthermore, although SDT has been extensively applied in educational contexts to enhance motivation and engagement in learning, and SRL has been shown to improve students’ literacy competencies (Anthonysamy et al., 2020; Chen et al., 2021; Z. Zhang et al., 2023), few studies have investigated the intersection of SDT, SRL, and AI literacy. This study aims to address this gap by examining how the psychological needs for autonomy, competence, and relatedness, as outlined in SDT, influence the development of students’ AI literacy through SRLSs.

### 2.3. This Study

**Figure 1:**
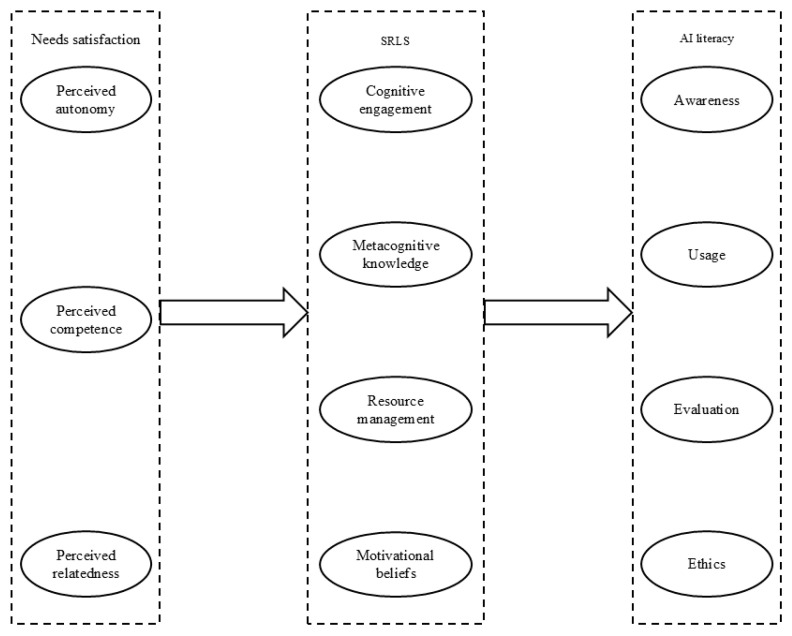
The hypothesized research model.

The research mentioned above has provided sufficient evidence to suggest there might be a relationship between university students’ need satisfaction and self-regulated learning strategies, and between self-regulated learning strategies and artificial intelligence literacy. This study seeks to examine the relationships between the three models and investigate whether stronger empirical connections exist between them at the dimensional level. Based on this, we propose the following hypotheses and present the research model, as illustrated in Figure 1.

**Hypothesis 1.**  *University students’ need satisfaction is related to their use of self-regulated learning strategies.*

**Hypothesis 2.**  *University students’ self-regulated learning strategies are related to their artificial intelligence literacy.*

**Hypothesis 3.**  *University students’ need satisfaction is related to their artificial intelligence literacy, mediated by self-regulated learning strategies.*

Figure 1 presents our research model.

## 3. Method

### 3.1. Context and Participants

A survey was conducted at a prominent university in China to test the research model. This university has been actively engaged in China’s “Artificial Intelligence +” strategy, promoting the integration of AI technology with higher education. It has held seminars and launched a special program for teaching reform to explore the application of AI in educational instruction, management, and research and talent development. In instructional settings, AI technology provides services such as question-answering and educational evaluation, assists in the automatic generation of lesson plans, evaluates teaching effectiveness, and supports an intelligent system for monitoring the teaching process. In administrative functions, AI is utilized for enrolment and course selection counseling, and a mental health counseling system has been developed. Additionally, in 2019, the university established the School of Artificial Intelligence as a talent cultivation hub, offering undergraduate and postgraduate programs in fields such as artificial intelligence and computer science.

The survey was conducted online through a link shared via email and posted on the university’s official website to ensure the confidentiality and security of the data. All personal details and responses were encrypted and utilized exclusively for the purposes of this study. The participants included 1056 university students, with a demographic breakdown of 237 males and 819 females, primarily undergraduates (87.8%). Additionally, a small number of students from higher diploma and postgraduate programs took part. The characteristics of the final sample size (N = 1056) are detailed in Table 1 for subsequent research.

### 3.2. Questionnaire and Test

This study used a structured questionnaire for data collection, divided into two parts. The first part gathered demographic information, such as participants’ age, gender, education level, and field of study. The second part consisted of a self-report questionnaire that assessed three key variables: SRLSs, AI literacy, and psychological need satisfaction (see Appendix A). All three variables were measured using a 5-point Likert scale.

#### 3.2.1. AI Literacy

AI literacy was assessed using a scale adapted from B. Wang et al. (2023), designed to measure students’ proficiency in applying AI technologies across four key dimensions: awareness, usage, evaluation, and ethics. Each dimension consisted of three items.

Awareness evaluates the ability to recognize and distinguish between AI-related concepts. A representative item is, “I can distinguish between smart devices and non-smart devices”. Usage measures how easily and frequently individuals engage with AI technologies, with items such as, “It is usually easy for me to learn to use a new AI application or product”. Evaluation examines the capacity to assess the functionality and limitations of AI technologies after using them. An example item is, “I can evaluate the capabilities and limitations of an AI application or product after using it for a while”. Finally, ethics evaluates students’ adherence to ethical principles in their use of AI, as demonstrated by, “I always comply with ethical principles when using AI applications or products”.

#### 3.2.2. Psychological Need Satisfaction

Psychological need satisfaction was assessed based on the framework of autonomy, competence, and relatedness, with each dimension consisting of four items. The items for autonomy and competence were adapted from Hew and Kadir (2016), while the items for relatedness were sourced from Furrer and Skinner (2003).

Autonomy refers to the sense of control over one’s learning processes, with a sample item being, “I feel like I can make a lot of input in deciding how I use the AI applications or products in learning”. Competence relates to the perceived ability to succeed in the learning process, with an example item like, “I think I am pretty good at learning with the AI applications or products”. Relatedness measures the extent to which students feel supported while learning, exemplified by, “When I learn with the AI applications or products, I feel supported”.

#### 3.2.3. SRLSs

SRLSs were assessed using the Motivated Strategies for Learning Questionnaire (MSLQ), a widely used instrument to measure self-regulated learning behaviors in university students, particularly in online educational contexts (Broadbent, 2017; Pintrich, 1991). The SRLS dimensions include resource management, motivational beliefs, metacognitive knowledge, and cognitive engagement.

Resource management assesses how students organize and manage learning resources, with five items, such as, “I collaborate with a group of friends to discuss AI technology”. Motivational beliefs measure students’ intrinsic motivation and goals for learning, with five items, including, “If I can, I want to do better than most of my classmates in my AI technology”. Metacognitive knowledge evaluates students’ awareness and regulation of their own learning processes, with four items, including, “If I get confused in learning AI technology, I use other methods to learn the technology”. Cognitive engagement reflects the degree of mental effort students invest in learning, with four items, such as, “When AI applications or products give materials, I develop my own ideas”.

## 4. Data Analysis and Results

In this research, partial least squares structural equation modeling (PLS-SEM) was chosen for its effectiveness in handling complex structural models. We employed SmartPLS 3.0 software to estimate both the measurement and structural models.

### 4.1. Measurement Model

The reliability, validity, correlations, and out loadings are shown in Table 2 and Table 3. The outer loadings, Cronbach’s alpha coefficient, and composite reliability (CR) values were above 0.70. The average variance extracted (AVE) values for each construct were above 0.50.

To assess the discriminant validity, the Fornell–Lacker criterion and cross-loadings were used in this study. The results showed that the square root of the AVE for each construct was greater than its correlation with the other constructs. In addition, the cross-loading analysis showed that each metric’s loading was significantly higher on its assigned structure than on the other structures.

### 4.2. Structural Model

PLS-SEM and Covariance-Based Structural Equation Modeling (CB-SEM) differ significantly in their methodologies. In CB-SEM, model fit indices are used to assess the overall match between the data and the model. In contrast, the main goal of PLS-SEM is to predict and explain the variance in the dependent variables, rather than to replicate the covariance matrix of the data. Consequently, conventional model fit indices are not applicable to PLS-SEM (Henseler et al., 2016).

The R^2^ values varied between 0.484 and 0.693 (cognitive engagement: R^2^ = 0.607; metacognitive knowledge: R^2^ = 0.599; resource management: R^2^ = 0.538; motivational beliefs: R^2^ = 0.632; awareness: R^2^ = 0.693; usage: R^2^ = 0.658; evaluation: R^2^ = 0.620; ethics: R^2^ = 0.484).

As shown in Table 4, regarding the relationship between need satisfaction and AI literacy, this study finds that perceived autonomy has a direct positive effect on cognitive engagement (β = 0.146, *p* < 0.001), metacognitive knowledge (β = 0.232, *p* < 0.001), motivational beliefs (β = 0.224, *p* < 0.001), and resource management (β = 0.206, *p* < 0.001). Similarly, perceived competence is found to have a direct positive effect on cognitive engagement (β = 0.345, *p* < 0.001), metacognitive knowledge (β = 0.282, *p* < 0.001), motivational beliefs (β = 0.269, *p* < 0.001), and resource management (β = 0.389, *p* < 0.001). Additionally, perceived relatedness has a direct positive effect on cognitive engagement (β = 0.367, *p* < 0.001), metacognitive knowledge (β = 0.347, *p* < 0.001), motivational beliefs (β = 0.389, *p* < 0.001), and resource management (β = 0.216, *p* < 0.001). Regarding the relationship between SRLSs and AI literacy, the evaluation results indicate that cognitive engagement significantly impacts awareness (β = 0.134, *p* < 0.01) and usage (β = 0.12, *p* < 0.01). However, its direct impact on ethics and evaluation is not significant. Metacognitive knowledge has a significant positive effect on awareness (β = 0.132, *p* < 0.01), ethics (β = 0.239, *p* < 0.001), and evaluation (β = 0.215, *p* < 0.001), whereas cognitive engagement does not significantly influence usage. Motivational beliefs have a significant positive impact on AI literacy, including awareness (β = 0.449, *p* < 0.001), ethics (β = 0.454, *p* < 0.001), evaluation (β = 0.308, *p* < 0.001), and usage (β = 0.47, *p* < 0.001). Resource management has a significant positive effect on awareness (β = 0.191, *p* < 0.01), usage (β = 0.209, *p* < 0.001), and evaluation (β = 0.251, *p* < 0.001), whereas cognitive engagement does not significantly influence ethics.

To further evaluate the importance of the indirect effects of predictor variables on university students’ AI literacy, this study conducted an analysis of the indirect effects. The data analysis results in Table 5 confirm the mediating role of SRLSs.

## 5. Discussion

This study aims to explore the complex dynamic relationships between students’ need satisfaction, the use of SRLSs, and AI literacy. After constructing the structural model, our analysis primarily focuses on testing three hypotheses to elucidate these relationships. Notably, the overall findings support the hypothesis that students’ need satisfaction positively predicts AI literacy, with SRLSs serving as a mediator.

Specifically, the first hypothesis examines the direct relationship between students’ need satisfaction and their engagement with SRLSs. Consistent with theoretical predictions, the results confirm that the satisfaction of all three psychological needs is essential in promoting college students’ use of SRLSs. This study fills the gap left by previous research, which has failed to empirically examine the connection between the two at the dimensional level. It explores in detail the role of satisfying students’ psychological needs in higher education, particularly in the context of artificial intelligence education, and provides empirical evidence that students’ need satisfaction predicts their use of SRLSs. This conclusion aligns with previous research that reports a positive relationship between students’ need satisfaction and their SRL (Xia et al., 2023a, 2023b). It further supports the argument that the satisfaction of psychological needs is a key factor in promoting positive learning behaviors and enhancing learning outcomes (Ryan & Deci, 2000), and extends the practical application of these theories to the use of emerging intelligent educational technologies.

Niemiec and Ryan (2009) emphasized that fulfilling students’ three basic psychological needs in an educational environment is essential for fostering intrinsic motivation and enhancing academic performance. Students are more likely to experience intrinsic motivation in an environment where they feel autonomous, which leads to greater participation in learning activities. In the context of AI education in China, many AI courses have progressively adopted project-based and self-directed learning modes, offering students increased autonomy and opportunities for decision-making, enabling them to explore AI-related content in depth based on their interests and career aspirations (R. Zheng et al., 2024). This autonomy encourages students to view learning as an active growth opportunity rather than a passive obligation, significantly enhancing learning engagement and the use of SRLSs. Indeed, the majority of studies have confirmed the positive impact of perceived autonomy (Schuitema et al., 2016) and teacher autonomy support (X. Bai & Gu, 2022) on students’ autonomous learning. This study explored the influence of different dimensions and found that perceived autonomy had a stronger impact on metacognitive knowledge (β = 0.232) and motivational beliefs (β = 0.224), likely because these dimensions are more directly related to students’ autonomous learning abilities and motivational regulation. These findings have important implications for designing supportive educational environments and courses.

As expected, the college students in this study were found to need perceived competence. For college students, believing in their ability to apply SRLSs is crucial for engaging in AI education. Students acquire relevant knowledge and skills through foundational courses such as computer programming, machine learning, and deep learning (Li et al., 2019). Perceived competence in this process can help students enhance their self-efficacy (Hirosawa et al., 2024), thereby influencing individual behavioral choices, effort levels, and persistence. When students feel capable of handling AI learning tasks, they are more likely to confidently apply advanced learning strategies, such as systematically using metacognitive engagement (β = 0.345) and effective resource management (β = 0.389).

A notable finding is the positive correlation between students’ perceived relatedness and their use of SRLSs. Although previous studies have demonstrated that relatedness can promote students’ online SRLSs (Zhou et al., 2021), the study by Xia et al. (2023b) found that relatedness did not significantly affect SRL through an artificial intelligence chatbot. The theory of mind in artificial intelligence (Langley et al., 2022) suggests that the interaction between AI and students is primarily cognitively driven, whereas (Picard, 2000) introduced the concept of affective computing, highlighting the importance of emotion in technological interaction. She argued that affective computing not only influences human emotional states but also significantly impacts learning outcomes. Particularly when AI possesses anthropomorphic characteristics, emotional interaction may play a crucial role in learning motivation and outcomes (Bölen et al., 2021; Pelau et al., 2021). In the field of intelligent education, AI systems’ emotional support for college students remains in the developmental stage. While some studies suggest that chatbots primarily provide cognitive support for learning (Kumar, 2021), the steady advancement of emotional AI research indicates that students’ interaction with anthropomorphic learning tools may also affect their emotional state, thereby further promoting autonomous learning behaviors. Therefore, further research is needed to explore how the anthropomorphic characteristics of artificial intelligence influence students’ learning behaviors.

The second hypothesis proposes a relationship between SRLSs and college students’ AI literacy. Although the general relationship between SRLSs and college students’ competency literacy has been established (Anthonysamy et al., 2020), further empirical evidence is required to determine whether these strategies play a significant role in acquiring AI literacy. The findings of this study not only confirm the positive relationship between SRLSs and college students’ AI literacy, as predicted by previous research, but, more importantly, position this relationship at a specific dimensional level.

**Figure 2:**
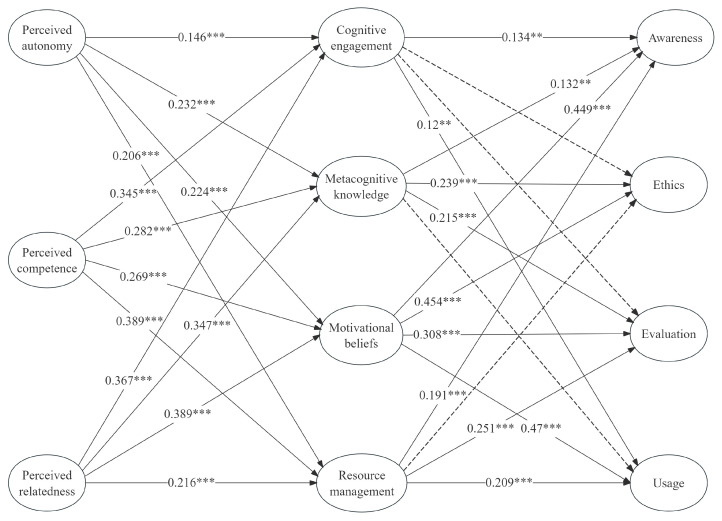
Result for the structural model. *** *p* < 0.001; ** *p* < 0.01.

Notably, in AI literacy, the ethics dimension had only two predictors, whereas the other dimensions had three or four. A review of the coefficient of determination for this dimension (see Figure 2) reveals that its explanatory power is relatively low. Motivational beliefs showed significant effects on all dimensions of AI literacy, including AI ethics, which constitutes a novel finding; to our knowledge, this has not been explored previously. Despite the absence of direct literature support, motivational beliefs remain an important mediator in fostering students’ ethics literacy. This may be because motivational beliefs help students maintain confidence and motivation in learning (Evans et al., 2024), thereby encouraging them to explore more complex and advanced content in AI technology. As found in the study by F. Wang et al. (2023), the expected value beliefs regarding learning AI are a crucial factor in determining whether students continue to explore the subject. Furthermore, research in related fields (such as motivation studies in STEM education) indicates that intrinsic motivation is essential for students’ critical thinking and deep learning (Schunk & Zimmerman, 2008). Therefore, we hypothesize that intrinsic motivational beliefs may play a similar role in AI ethics education. However, the results of this study must be verified through larger-scale empirical research. Moreover, metacognitive knowledge also exerts a significant positive impact on the ethical dimension. Upadhyay (2024) found that AI developers’ understanding of ethical issues influences how they address AI ethical concerns in practice, which aligns with this result. This study suggests that students with strong metacognitive knowledge are more likely to be aware of their own thinking processes and can analyze problems from multiple perspectives. This reflective ability enables them to make more rational and comprehensive judgments when confronted with ethical dilemmas.

This study identified connections between motivational beliefs, resource management, and the three dimensions of AI literacy, excluding AI ethics. Motivational beliefs positively impact the usage dimension, consistent with the findings of Kim et al. (2024). Furthermore, it positively influences awareness, aligning with the results of Yin et al. (2024). As previously noted, students’ motivational beliefs, such as intrinsic motivation, can enhance their interest and cognitive engagement with the learning content (Schunk & Zimmerman, 2008). When students are highly motivated, they are more inclined to explore and comprehend new technologies, including AI, thereby enhancing their basic understanding and awareness of AI. In the context of AI application, students’ motivation drives them to actively engage in learning activities, and highly motivated students tend to use AI technology more frequently and confidently. Students’ motivational beliefs also influence their ability to critically evaluate AI tools and applications. This represents a novel insight. Motivated students are more likely to conduct a comprehensive analysis of the advantages, disadvantages, and functional limitations of AI, which aids them in making more accurate judgments. Effective resource management also enables students to optimize the learning process and enhance their learning efficiency (Han et al., 2024), thereby improving their performance across various dimensions of AI literacy. As demonstrated, students proficient in resource management are able to use their time and energy more efficiently, exhibiting higher engagement and awareness in AI learning, making the connection with students’ awareness, use, and evaluation of AI comprehensible. However, the lack of a connection between resource management and AI ethics is noteworthy. This finding further underscores the complexity of ethical awareness and suggests that cultivating AI ethical literacy necessitates a dedicated educational approach, distinct from other learning strategies.

Beyond the ethics dimension, this study also demonstrates that metacognitive knowledge significantly influences the awareness and evaluation dimensions, a finding in line with Pintrich (2002). This implies that metacognitive knowledge enables students to identify and understand the concepts and skills involved in learning. For instance, students can recognize how AI tools assist in learning and become aware of their applications and societal impact. Furthermore, metacognitive knowledge aids students in evaluating and critically analyzing the effectiveness, advantages, disadvantages, and potential limitations of AI tools. Through self-monitoring and adjustment strategies, students are able to identify issues arising from the use of AI technology and make appropriate adjustments.

Another notable finding is the association between cognitive engagement and the awareness and use dimensions. Cognitive engagement positively influences the awareness dimension, a novel finding to our knowledge. This conclusion is supported by deep learning theory, which asserts that the greater students’ cognitive engagement, the deeper their understanding of the learning content (Marton & Säljö, 1976). Active thinking and reflection assist students in constructing a more stable knowledge structure, thus enhancing their cognitive level in AI learning. Moreover, cognitive engagement positively influences the use dimension, aligning with Li et al. (2024). Greater cognitive engagement increases students’ confidence in using AI tools, leading to more active use and effective problem-solving in learning. While cognitive engagement significantly impacts the cognition and use dimensions of AI, it has no significant effect on the ethical and evaluation dimensions of AI literacy. According to critical thinking theory, although cognitive engagement significantly impacts the depth of understanding, critical evaluation and ethical judgment rely more on social and emotional factors, such as social responsibility, moral perception, and ethical education (Elder & Paul, 2020). This implies that students require more ethical education and social context support, rather than relying solely on cognitive engagement, to understand the ethical issues surrounding AI.

The third hypothesis posits that students’ need satisfaction mediates the relationship between SRLSs and AI literacy. The results indicate that the relationship between students’ need satisfaction and AI literacy is significant at the dimensional level, strongly confirming the structural hypothesis. The findings are particularly significant as they reveal that while all four SRLSs (resource management, motivational beliefs, metacognitive knowledge, and cognitive engagement) contribute to fostering awareness, three SRLSs (resource management, motivational beliefs, and cognitive engagement) facilitate AI usage, and only three SRLSs (motivational beliefs, metacognitive knowledge, and cognitive engagement) mediate the relationship between need satisfaction and evaluation. Notably, the ethics dimension of AI literacy was the least affected by SRLSs, with only two strategies—motivational beliefs and metacognitive knowledge—mediating the relationship.

These findings corroborate existing literature that underscores the positive, indirect relationship between students’ need satisfaction and AI literacy via SRLSs (Anthonysamy et al., 2020; Chen et al., 2021; Z. Zhang et al., 2023). Previous studies have emphasized that fulfilling students’ basic psychological needs (e.g., autonomy, competence, and relatedness) is crucial for enhancing their motivation and engagement (Olafsen et al., 2018; Sicard et al., 2024), which, in turn, facilitates their development of AI literacy. This study extends these findings by identifying specific SRLSs that mediate the relationship between need satisfaction and the distinct dimensions of AI literacy, offering a more nuanced understanding of the underlying mechanisms.

Moreover, this research underscores the complexity of how psychological need satisfaction interacts with SRLSs in fostering AI literacy. The differentiated impact across various dimensions of AI literacy suggests that the development of awareness, usage, evaluation, and ethics may require tailored strategies that account for the distinct types of SRLS. The finding that motivational beliefs and metacognitive knowledge are particularly influential in shaping ethical considerations related to AI further underscores the importance of cultivating these aspects in educational settings. These findings contribute to the expanding body of literature on the role of SRL in AI education and offer actionable insights for designing effective educational interventions that enhance students’ AI literacy.

## 6. Implications

### 6.1. Theoretical Implications

The findings align with SDT, confirming that perceived autonomy, competence, and relatedness foster intrinsic motivation, which enhances students’ application of SRLSs. This underscores the importance of satisfying students’ basic psychological needs in cultivating effective learning behaviors, particularly in demanding fields like AI education. Furthermore, as previously mentioned, the founders of SDT suggested that SDT research should focus on the design of learning technologies (Ryan & Deci, 2020). This study extends the scope of SDT research into emerging technologies and advanced technical education by establishing a link between need satisfaction and AI literacy.

This study enriches the existing literature on SRLSs by demonstrating its critical role in fostering AI literacy. While prior research has explored the role of SRLSs in academic performance (Cetintav & Yilmaz, 2023) and general digital literacy (Anthonysamy et al., 2020), this study reveals its multidimensional influence on AI literacy, including awareness, ethics, evaluation, and usage. The findings emphasize that specific SRLS dimensions, such as motivational beliefs and metacognitive knowledge, are particularly effective in developing key aspects of AI literacy, such as moral reasoning and critical evaluation. The lack of a significant relationship between resource management and AI ethical awareness highlights the complexity of fostering ethical literacy in AI education. This provides a nuanced understanding of how SRLSs support students’ holistic development in technical fields.

The results of this study contribute to advancing the theoretical understanding of AI literacy, particularly at the intersection of psychological, educational, and technological domains. First, the findings align with SDT, confirming that perceived autonomy, competence, and relatedness foster intrinsic motivation, thereby enhancing students’ use of SRLS. This highlights the critical role of fulfilling students’ basic psychological needs in cultivating effective learning behaviors, especially in challenging fields like AI education. Notably, while SDT has been widely applied in various educational contexts, this study expands its relevance by applying it to the rapidly evolving field of AI education. By establishing a connection between need satisfaction and AI literacy, this study not only corroborates but also extends the scope of SDT research into emerging technologies and advanced technical education. Incorporating SDT into AI education offers a novel theoretical perspective on how intrinsic motivation can be harnessed to improve learning outcomes in this highly specialized area.

### 6.2. Practical Implications

The rapid expansion of AI education in China necessitates a more focused approach to integrating AI literacy into higher education. The findings of this study offer key insights for educators and curriculum designers in effectively fostering AI literacy among students.

For educators, this implies that curricula should not only address students’ cognitive learning needs but also their psychological needs. Instructional strategies should prioritize the promotion of SRLSs by incorporating hands-on project-based learning, such as AI-based applications or coding challenges that encourage experimentation. Additionally, reflective activities—such as group discussions or journaling—can help students enhance their metacognitive awareness by critically assessing their learning processes and identifying areas for improvement. By doing so, students can develop the necessary skills to navigate AI learning challenges independently. Teachers should also provide motivational support through individualized feedback and encouragement, helping students build self-confidence in tackling complex AI topics.

Curriculum designers should ensure that AI education emphasizes a balanced approach—developing both technical proficiency and ethical understanding. This is especially important in light of national policies like the “China Education Modernization 2035” plan, which highlight the need to promote self-efficacy, self-directed learning, and the holistic development of students. For instance, integrating case studies on AI ethics, such as the use of AI in surveillance or decision-making, can help students critically examine the ethical implications of AI technologies.

Moreover, universities and educational institutions must cultivate environments that support collaboration and relationship-building between students, peers, and instructors. Such environments not only enhance students’ sense of connectedness but also enable them to engage in collaborative learning necessary to develop complex cognitive and learning strategies. For example, creating AI-focused student clubs or organizing hackathons can foster a sense of community and collaboration. Institutions should also prioritize the inclusion of AI ethics modules and promote interdisciplinary approaches to AI education. Courses that combine AI with subjects like philosophy, sociology, or law can provide students with a broader understanding of the social and ethical implications of AI. This will ensure they are well prepared for responsible participation in a rapidly evolving technological landscape.

## 7. Limitations and Suggestions for Future Research

A limitation of this study is that it does not fully address the actual extent of AI use in students’ learning processes. Although the university selected for this study actively promotes AI and integrates it into educational practices, the research does not clarify how extensively AI is used by students in their daily learning activities. The integration of AI tools and resources into the curriculum may vary significantly across courses, disciplines, and teaching styles, potentially influencing the results. Future research should investigate how the actual use of AI in learning environments interacts with students’ engagement in SRLSs and the development of AI literacy. Furthermore, understanding the specific types of AI applications used by students—such as AI-powered educational platforms, virtual assistants, and other learning tools—would offer a more nuanced understanding of how AI impacts students’ learning experiences and the development of their AI literacy.

Following an extensive survey of university undergraduates, we identified several key findings. However, it is important to acknowledge that the results may not fully represent all universities, as AI technology is currently implemented in only a limited number of institutions. Moreover, the influence of Chinese educational culture on students’ AI literacy may limit the generalizability of these findings to other countries or educational systems. Consequently, future research should consider a broader range of colleges and universities, both within China and internationally, to ensure a more comprehensive and representative dataset. It would also be beneficial to explore how different educational cultures might shape students’ development of AI literacy.

This study primarily relied on self-reported questionnaires for quantitative analysis to assess students’ ability levels in the areas of psychological needs, SRLSs, and AI literacy, rather than their perceptions of these factors. However, since self-report instruments are susceptible to biases that can influence data accuracy, this may limit the reliability of the findings. As questionnaires primarily capture students’ subjective reports, they may not fully reflect their actual abilities in these areas. Future studies should incorporate objective academic performance indicators, such as student grades or assessment scores, alongside self-reported data. This approach would facilitate a more comprehensive analysis of how AI literacy and SRLS influence actual learning outcomes. By triangulating subjective self-reports with objective performance measures, researchers can enhance the validity and generalizability of their findings. Furthermore, the use of longitudinal or quasi-experimental designs could help establish causal relationships and track changes over time.

A substantial proportion of our participants came from the humanities and social sciences, which may have affected the comprehensiveness of the study. Given their limited exposure to AI technologies, these students might have distinct concerns regarding the impact of AI on their disciplines compared to students from other fields. Furthermore, the sample was predominantly female, which may introduce a gender bias and influence the generalizability of the findings. This gender imbalance could affect the interpretation of AI literacy levels, as prior research suggests that gender may influence technology engagement and perceptions (Cai et al., 2017). Therefore, future studies should strive to include a more diverse demographic, balancing both academic disciplines and gender, to enhance the depth, representativeness, and generalizability of the findings.

Another limitation of this study was the inability to differentiate participants based on their year of study. Given that students at different stages of their studies may have exhibited varying levels of need satisfaction, SRLSs, and AI literacy, a more detailed breakdown could have yielded additional insights. However, in designing the survey, we primarily focused on the participants’ level of degree and did not account for the year of study as a potential influencing factor. Additionally, to maintain clarity and conciseness in the demographic information table, this variable was not included in the survey.

Future research should investigate how students’ progression through their studies influences their AI literacy and SRLSs. A longitudinal or cross-sectional approach that examines differences between first-year and final-year students could provide a more nuanced understanding of how these factors evolve throughout higher education.

## 8. Conclusions

AI technologies are transforming professional and personal domains, necessitating a foundational understanding of AI literacy among university students to prepare them for future workplace challenges. This study, grounded in SDT, sheds light on the psychological and behavioral determinants of AI literacy, focusing on the role of need satisfaction and SRLSs. The findings highlight the critical influence of perceived autonomy, competence, and relatedness in shaping students’ engagement with SRLSs and, in turn, enhancing their AI literacy. Specifically, motivational beliefs, metacognitive knowledge, resource management, and cognitive engagement emerged as key mediators in this relationship, underscoring the importance of both psychological needs and effective learning strategies in fostering AI literacy. These insights contribute to the understanding of how university students navigate the complexities of AI education, providing both theoretical validation and practical frameworks for educators and policymakers.

## Figures and Tables

**Table 1:** Characteristics of the sample (N = 1056).

Profile	Category	Frequency	Percentage (%)
Gender	Male	237	22.4
	Female	819	77.5
Age	18–22	889	84.1
	23–27	121	11.4
	27–31	15	1.4
	≥32	31	2.9
Level of degree	Undergraduate	928	87.8
	Master	120	11.3
	PhD	7	0.7
Major	Education	467	44.2
	Literature	253	23.9
	Science	86	8.1
	Management	43	4.0
	Other	207	19.6

Note: The ‘Other’ category includes disciplines that do not fit neatly into the predefined categories, such as engineering, fine arts, law, and medicine, as well as interdisciplinary fields like environmental science, data science, and cognitive science.

**Table 2:** Reliability and validity metrics.

Construct	Cronbach’s Alpha	CR	AVE
Awareness	0.852	0.91	0.772
Ethics	0.903	0.939	0.837
Evaluation	0.874	0.923	0.799
Usage	0.865	0.917	0.787
Cognitive engagement	0.874	0.913	0.725
Motivational beliefs	0.906	0.93	0.726
Metacognitive knowledge	0.89	0.924	0.752
Resource management	0.826	0.878	0.59
Perceived autonomy	0.866	0.909	0.714
Perceived competence	0.852	0.9	0.693
Perceived relatedness	0.905	0.934	0.778

**Table 3:** Discriminant validity evaluation.

**Fornell–Lacker Criterion**											
	AW	CE	ET	EV	MB	MK	RM	US	PA	PC	PR
AW	**0.879**										
CE	0.724	**0.851**									
ET	0.591	0.603	**0.915**								
EV	0.768	0.685	0.653	**0.894**							
MB	0.8	0.757	0.67	0.735	**0.852**						
MK	0.729	0.836	0.628	0.711	0.764	**0.867**					
RM	0.737	0.732	0.55	0.716	0.779	0.743	**0.768**				
US	0.844	0.695	0.559	0.769	0.785	0.694	0.722	**0.887**			
PA	0.644	0.625	0.501	0.596	0.664	0.654	0.619	0.633	**0.845**		
PC	0.705	0.728	0.497	0.662	0.725	0.711	0.699	0.716	0.706	**0.832**	
PR	0.662	0.723	0.563	0.636	0.739	0.711	0.644	0.662	0.641	0.762	**0.882**
**Cross-Loadings**											
	AW	CE	ET	EV	MB	MK	PA	PC	PR	RM	US
AW1	**0.873**	0.61	0.542	0.686	0.689	0.62	0.556	0.557	0.55	0.62	0.703
AW2	**0.901**	0.657	0.549	0.678	0.736	0.659	0.575	0.659	0.635	0.655	0.748
AW3	**0.862**	0.641	0.467	0.661	0.683	0.641	0.565	0.641	0.558	0.667	0.774
CE1	0.626	**0.85**	0.521	0.597	0.664	0.71	0.541	0.661	0.666	0.591	0.588
CE2	0.586	**0.839**	0.499	0.535	0.614	0.657	0.471	0.606	0.595	0.62	0.569
CE3	0.606	**0.868**	0.52	0.598	0.669	0.731	0.555	0.613	0.635	0.628	0.615
CE4	0.646	**0.85**	0.513	0.601	0.632	0.747	0.556	0.598	0.565	0.653	0.595
ET1	0.562	0.558	**0.91**	0.625	0.631	0.586	0.459	0.458	0.525	0.531	0.529
ET2	0.531	0.551	**0.92**	0.606	0.607	0.579	0.476	0.46	0.529	0.488	0.51
ET3	0.529	0.546	**0.915**	0.559	0.599	0.559	0.441	0.445	0.49	0.49	0.496
EV1	0.692	0.615	0.55	**0.886**	0.638	0.636	0.526	0.6	0.569	0.653	0.686
EV2	0.683	0.607	0.612	**0.909**	0.671	0.645	0.549	0.601	0.589	0.65	0.69
EV3	0.685	0.616	0.587	**0.887**	0.662	0.626	0.523	0.573	0.547	0.616	0.686
MB1	0.678	0.628	0.542	0.622	**0.843**	0.626	0.586	0.636	0.603	0.686	0.69
MB2	0.67	0.636	0.477	0.597	**0.845**	0.623	0.547	0.639	0.621	0.678	0.665
MB3	0.696	0.692	0.622	0.657	**0.879**	0.707	0.58	0.642	0.665	0.697	0.689
MB4	0.699	0.66	0.651	0.649	**0.852**	0.68	0.577	0.585	0.637	0.633	0.653
MB5	0.664	0.608	0.554	0.604	**0.84**	0.615	0.537	0.585	0.62	0.626	0.646
MK1	0.66	0.756	0.535	0.631	0.66	**0.882**	0.574	0.626	0.605	0.661	0.635
MK2	0.634	0.724	0.529	0.592	0.658	**0.881**	0.571	0.617	0.611	0.662	0.611
MK3	0.657	0.742	0.532	0.639	0.675	**0.891**	0.584	0.635	0.617	0.67	0.624
MK4	0.576	0.677	0.587	0.604	0.658	**0.813**	0.538	0.586	0.635	0.584	0.535
PA1	0.572	0.566	0.441	0.566	0.61	0.577	**0.824**	0.591	0.548	0.579	0.551
PA2	0.56	0.497	0.357	0.5	0.531	0.521	**0.839**	0.627	0.509	0.519	0.567
PA3	0.543	0.516	0.419	0.48	0.545	0.55	**0.88**	0.614	0.547	0.511	0.538
PA4	0.495	0.524	0.471	0.461	0.55	0.556	**0.835**	0.553	0.558	0.474	0.48
PC1	0.614	0.553	0.313	0.523	0.544	0.53	0.594	**0.796**	0.521	0.603	0.632
PC2	0.572	0.629	0.483	0.57	0.626	0.641	0.632	**0.837**	0.684	0.573	0.586
PC3	0.528	0.6	0.424	0.517	0.588	0.577	0.515	**0.829**	0.678	0.55	0.53
PC4	0.634	0.637	0.427	0.588	0.649	0.613	0.607	**0.866**	0.65	0.602	0.637
PR1	0.588	0.653	0.546	0.566	0.673	0.664	0.569	0.68	**0.892**	0.573	0.569
PR2	0.609	0.632	0.446	0.578	0.648	0.625	0.591	0.687	**0.868**	0.598	0.614
PR3	0.556	0.621	0.479	0.547	0.628	0.586	0.536	0.661	**0.881**	0.558	0.572
PR4	0.583	0.645	0.514	0.552	0.655	0.632	0.565	0.662	**0.888**	0.544	0.582
RM1	0.543	0.545	0.377	0.545	0.544	0.552	0.45	0.531	0.455	**0.801**	0.542
RM2	0.584	0.552	0.371	0.569	0.555	0.548	0.468	0.551	0.474	**0.748**	0.576
RM3	0.536	0.546	0.322	0.506	0.511	0.506	0.417	0.523	0.406	**0.774**	0.533
RM4	0.591	0.61	0.562	0.604	0.678	0.673	0.549	0.548	0.569	**0.789**	0.58
RM5	0.568	0.549	0.457	0.514	0.687	0.557	0.479	0.528	0.553	**0.728**	0.537
US1	0.762	0.62	0.448	0.649	0.685	0.618	0.578	0.641	0.56	0.652	**0.903**
US2	0.757	0.601	0.417	0.683	0.651	0.583	0.535	0.633	0.545	0.636	**0.887**
US3	0.728	0.627	0.613	0.713	0.746	0.643	0.57	0.632	0.652	0.633	**0.87**

Note: 1. The bold numbers in Table 3 represent the PLS loadings in their construct. 2. AW = awareness; US = usage; EV = evaluation; ET = ethics; CE = cognitive engagement; MB = motivational beliefs; MK = metacognitive knowledge; RM = resource management; PA = perceived autonomy; PC = perceived competence; PR = perceived relatedness. These abbreviations also apply to other tables.

**Table 4:** Model path results.

Path	β	*p*-Value	Result
Perceived autonomy → Cognitive engagement	0.146	***	Yes
Perceived autonomy → Metacognitive knowledge	0.232	***	Yes
Perceived autonomy → Motivational beliefs	0.224	***	Yes
Perceived autonomy → Resource management	0.206	***	Yes
Perceived competence → Cognitive engagement	0.345	***	Yes
Perceived competence → Metacognitive knowledge	0.282	***	Yes
Perceived competence → Motivational beliefs	0.269	***	Yes
Perceived competence → Resource management	0.389	***	Yes
Perceived relatedness → Cognitive engagement	0.367	***	Yes
Perceived relatedness → Metacognitive knowledge	0.347	***	Yes
Perceived relatedness → Motivational beliefs	0.389	***	Yes
Perceived relatedness → Resource management	0.216	***	Yes
Cognitive engagement → Awareness	0.134	**	Yes
Cognitive engagement → Ethics	n.s.	n.s.	No
Cognitive engagement → Evaluation	n.s.	n.s.	No
Cognitive engagement → Usage	0.12	**	Yes
Metacognitive knowledge → Awareness	0.132	**	Yes
Metacognitive knowledge → Ethics	0.239	***	Yes
Metacognitive knowledge → Evaluation	0.215	***	Yes
Metacognitive knowledge → Usage	n.s.	n.s.	No
Resource management → Awareness	0.191	***	Yes
Resource management → Ethics	n.s.	n.s.	No
Resource management → Evaluation	0.251	***	Yes
Resource management → Usage	0.209	***	Yes
Motivational beliefs → Awareness	0.449	***	Yes
Motivational beliefs → Ethics	0.454	***	Yes
Motivational beliefs → Evaluation	0.308	***	Yes
Motivational beliefs → Usage	0.47	***	Yes

Note: *** *p* < 0.001; ** *p* < 0.01; n.s. = not significant.

**Table 5:** Indirect effects.

Path	Specific Path	Path Coefficient	*p*-Value
PA → AW		0.19	***
	PA → CE → AW	0.019	*
	PA → MK → AW	0.031	**
	PA → RM → AW	0.039	***
	PA → MB → AW	0.101	***
PA → US		0.184	***
	PA → CE → US	0.018	*
	PA → MK → US	n.s.	n.s.
	PA → RM → US	0.043	***
	PA → MB → US	0.105	***
PA → EV		0.184	***
	PA → CE → EV	n.s.	n.s.
	PA → MK → EV	0.05	**
	PA → RM → EV	0.052	***
	PA → MB → EV	0.069	***
PA → ET		0.161	***
	PA → CE → ET	n.s.	n.s.
	PA → MK → ET	0.055	**
	PA → RM → ET	n.s.	n.s.
	PA → MB → ET	0.102	***
PC → AW		0.279	***
	PC → CE → AW	0.046	**
	PC → MK → AW	0.037	**
	PC → RM → AW	0.074	***
	PC → MB → AW	0.121	***
PC → US		0.272	***
	PC → CE → US	0.041	*
	PC → MK → US	n.s.	n.s.
	PC → RM → US	0.081	***
	PC → MB → US	0.127	***
PC → EV		0.272	***
	PC → CE → EV	n.s.	n.s.
	PC → MK → EV	0.061	***
	PC → RM → EV	0.098	***
	PC → MB → EV	0.083	***
PC → ET		0.203	***
	PC → CE → ET	n.s.	n.s.
	PC → MK → ET	0.067	***
	PC → RM → ET	n.s.	n.s.
	PC → MB → ET	0.122	***
PR → AW		0.311	***
	PR → CE → AW	0.049	**
	PR → MK → AW	0.046	**
	PR → RM → AW	0.041	***
	PR → MB → AW	0.175	***
PR → US		0.3	***
	PR → CE → US	0.044	**
	PR → MK → US	n.s.	n.s.
	PR → RM → US	0.045	***
	PR → MB → US	0.183	***
PR → EV		0.281	***
	PR → CE → EV	n.s.	n.s.
	PR → MK → EV	0.075	***
	PR → RM → EV	0.054	***
	PR → MB → EV	0.12	***
PR → ET		0.284	***
	PR → CE → ET	n.s.	n.s.
	PR → MK → ET	0.083	**
	PR → RM → ET	n.s.	n.s.
	PR → MB → ET	0.177	***

Note: *** *p* < 0.001; ** *p* < 0.01; * *p* < 0.05; n.s. = not significant.

## Data Availability

The datasets used in the current study are available from the corresponding author upon reasonable request.

## Appendix A. The Instruments

**Psychological Need Satisfaction**	
Perceived autonomy	1. I feel like I can make a lot of input in deciding how I use the AI applications or products in learning. 2. I feel a sense of freedom when using the AI applications or products. 3. I have many opportunities with the AI applications or products to decide for myself how to learn. 4. I have a say regarding what input I want to learn with AI applications or products.
Perceived competence	5. I think I am pretty good at learning with the AI applications or products. 6. I have been able to learn interesting new knowledge with the AI applications or products. 7. I feel a sense of accomplishment from learning with the AI applications or products. 8. When I am using AI applications or products I often do not feel very capable ^R^
Perceived relatedness	9. When I learn with the AI applications or products, I feel supported. 10. When I learn with the AI applications or products, I feel comfortable. 11. When I learn with the AI applications or products, I feel important. 12. When I learn with the AI applications or products, I feel valued.
**Self-regulated learning strategies**	
Cognitive engagement	13. When I learn about AI technology, I use the existing knowledge that I have. 14. I write my own notes or outline important concepts while using AI applications or products. 15. I ask myself whether the materials provided by the AI application or product are useful for my task. 16. When AI applications or products give materials, I develop my own ideas.
Metacognitive knowledge	17. When I use AI applications or products to solve a problem, I analyse it first before starting it (e.g., read the questions properly, know the expected outcomes and objectives). 18. I set some standards (what I need to achieve) for my AI technology. 19. When doing tasks using AI applications or products, I question myself to help understand the task better. 20. If I get confused in learning AI technology, I use other methods to learn the technology.
Resource management	21. I collaborate with a group of friends to discuss AI technology. 22. If I have trouble learning AI technology, I try to figure it out on my own without asking for help. 23. I email or talk to my lecturer to clarify what I don’t understand about AI technology. 24. When I can’t understand AI technology, I get feedback from other students or social media to help me. 25. I work hard to learn AI technology even though I don’t like it.
Motivational beliefs	26. I am confident I can use AI technology well. 27. I am interested to learn from any AI applications or products because I believe it is important. 28. I believe I will use my AI knowledge in other subjects. 29. I work hard to master new things, for example, learning a new software. 30. If I can, I want to do better than most of my classmates in my AI technology.
**Artificial Intelligence Literacy**	
Awareness	31. I can distinguish between smart devices and non-smart devices. 32. I do not know how AI technology can help me. ^R^ 33. I can identify the AI technology employed in the applications and products I use.
Usage	34. I can skilfully use AI applications or products to help me with my daily work. 35. It is usually easy for me to learn to use a new AI application or product. 36. I can use AI applications or products to improve my work efficiency.
Evaluation	37. I can evaluate the capabilities and limitations of an AI application or product after using it for a while. 38. I can choose a proper solution from various solutions provided by a smart agent. 39. I can choose the most appropriate AI application or product from a variety for a particular task.
Ethics	40. I always comply with ethical principles when using AI applications or products. 41. I am alert to privacy and information security issues when using AI applications or products. 42. I am always alert to the abuse of AI technology.
